# Development and single‐particle analysis of hybrid extracellular vesicles fused with liposomes using viral fusogenic proteins

**DOI:** 10.1002/2211-5463.13406

**Published:** 2022-04-30

**Authors:** Raga Ishikawa, Shosuke Yoshida, Shin‐ichi Sawada, Yoshihiro Sasaki, Kazunari Akiyoshi

**Affiliations:** ^1^ 12918 Department of Polymer Chemistry Graduate School of Engineering Kyoto University Japan; ^2^ Present address: 12918 Department of Environmental Engineering Graduate School of Engineering Kyoto University Katsura, Nishikyo‐ku Kyoto Japan; ^3^ Present address: Division of Biological Science Nara Institute of Science and Technology Ikoma Nara Japan

**Keywords:** baculovirus‐expression system, extracellular vesicle, liposome, membrane fusion, single‐particle analysis, viral fusogenic protein

## Abstract

Extracellular vesicles (EVs) have potential biomedical applications, particularly as a means of transport for therapeutic agents. There is a need for rapid and efficient EV‐liposome membrane fusion that maintains the integrity of hybrid EVs. We recently described Sf9 insect cell‐derived EVs on which functional membrane proteins were presented using a baculovirus‐expression system. Here, we developed hybrid EVs by membrane fusion of small liposomes and EVs equipped with baculoviral fusogenic proteins. Single‐particle analysis of EV‐liposome complexes revealed controlled introduction of liposome components into EVs. Our findings and methodology will support further applications of EV engineering in biomedicine.

## Graphical abstract

We developed hybrid extracellular vesicles by membrane fusion of small liposomes and insect cell‐derived extracellular vesicles equipped with baculoviral fusogenic proteins. Single‐particle analysis of extracellular vesicle‐liposome complexes revealed controlled introduction of liposome components into extracellular vesicles via baculoviral protein‐mediated membrane fusion.

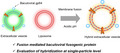

**Abbreviations**
BV: budded virus
CFSE: 5‐ or 6‐(*N*‐succinimidyloxycarbonyl)fluorescein 3′,6′‐diacetate
Cx43: Connexin 43
Cy5‐DOPE: 1,2‐Dioleoyl‐*sn*‐glycero‐3‐phosphoethanolamine‐*N*‐(Cyanine 5)
DOPC: 1,2‐Dioleoyl‐*sn*‐glycero‐3‐phosphocholine
DOPS: 1,2‐dioleoyl‐*sn*‐glycero‐3‐phospho‐l‐serine
EGFP: enhanced green fluorescence protein
EV: extracellular vesicle
FRET: fluorescence resonance energy transfer
IFC: imaging flow cytometry
NBD: 7‐Nitro‐2‐1,3‐benzoxadiazol‐4‐yl
NBD‐DOPE: 1,2‐Dioleoyl‐*sn*‐glycero‐3‐phosphoethanolamine‐N‐(7‐nitro‐2‐1,3‐benzoxadiazol‐4‐yl)
PD‐1: programmed cell death 1
Rho‐DOPE: 1,2‐Dioleoyl‐*sn*‐glycero‐3‐phosphoethanolamine‐*N*‐(lissamine rhodamine B sulfonyl)
TEM: transmission electron microscopy

Extracellular vesicles (EVs) are cell membrane‐derived vesicles that play important roles in cell‐cell communications (e.g. immune responses and cancer metastasis) by delivering signal transduction molecules from parent cells to target cells [1, 2, 3, 4]. Thus, EVs are presumably useful in biomedical applications such as cancer diagnosis, vaccination, and regenerative medicine [5]. In particular, there is growing interest in engineered EVs for use in drug delivery because of their abilities to effectively transport therapeutic agents [5].

Genetic engineering techniques are often used to control EV functions. By gene transfection modifications of parent cells, target proteins or peptides can be presented on released EVs, whereas bioactive cargos (e.g. nucleic acids or proteins) are loaded inside the EVs [6]. These engineered EVs have been used in applications including drug delivery [7], vaccines [8], and biosensors [9]. In addition to genetic engineering methods, EVs have been modified by chemical and physical complexation with functional biomaterials [10]. For example, pH responsiveness [11], surface charge [12, 13], magnetic‐responsiveness [14], and targeting ability [15] have been added to EVs.

Liposomes are excellent candidates for EV modification because liposomal components (e.g. functional lipids or encapsulated drugs) can be introduced to EVs through membrane fusion. EVs and liposomes have been fused by simple mixing [16], freeze‐thaw processes [17], and poly(ethylene glycol) addition [18]. However, the fusion efficiency is sometimes low and there are concerns involving damage to hybrid EV membranes or contamination with exogenous polymer. Thus, there is a need for a more rapid and efficient membrane fusion pathway that does not compromise the integrity of hybrid EVs. Furthermore, it is important to analyze the fusion process at the single‐particle level because the membrane composition of EVs is heterogeneous and the fusion behavior is likely to be different for each EV [19, 20].

Our research focuses on viral fusogenic proteins to generate EV‐liposome hybrids. Envelope viruses infect host cells by fusogenic proteins that have rapid environmentally responsive fusion functions (e.g. influenza hemagglutinin and human T‐cell leukemia virus envelope protein) [21]. We recently developed Sf9 insect cell‐derived EVs on which functional membrane proteins were presented using a baculovirus‐expression system [22]. Programmed cell death 1 (PD‐1) membrane proteins were expressed on the EV surface while maintaining the ability to bind to ligand proteins and ligand‐expressing cancer cells. In addition, the baculoviral envelope protein gp64 was expressed on the surface of PD‐1‐expressing EVs (PD‐1 EVs). Gp64 is known to exhibit a membrane fusion function under acidic conditions and is critical in baculovirus infection and budding [23]. The gp64 present in virus‐like particles from Sf9 insect cells is able to mediate membrane fusion with giant liposomes [24, 25, 26]. Although this fusion method is useful for the preparation of hybrid proteoliposomes for biomedical applications [25, 26], the fusion behavior is not fully understood at single‐particle level. Here, we report a detailed evaluation of pH‐responsive fusion of small liposomes and Sf9‐derived EVs equipped with fusogenic gp64 by lipid mixing and single‐particle analyses.

## Materials and methods

### Materials

The Sf9 insect cell line, derived from the fall armyworm *Spodoptera frugiperda*, was purchased from Invitrogen (Carlsbad, CA, USA). Mouse monoclonal anti‐baculovirus glycoprotein gp64 IgG antibody (AcV5, sc‐65499) was purchased from Santa Cruz Biotechnology (Dallas, TX, USA). 1,2‐Dioleoyl‐*sn*‐glycero‐3‐phosphocholine (DOPC), 1,2‐dioleoyl‐sn‐glycero‐3‐phospho‐l‐serine (DOPS), 1,2‐dioleoyl‐*sn*‐glycero‐3‐phosphoethanolamine‐*N*‐(7‐nitro‐2‐1,3‐benzoxadiazol‐4‐yl) (NBD‐DOPE), 1,2‐dioleoyl‐*sn*‐glycero‐3‐phosphoethanolamine‐*N*‐(lissamine rhodamine B sulfonyl) (Rho‐DOPE), and 1,2‐dioleoyl‐sn‐glycero‐3‐phosphoethanolamine‐*N*‐(Cyanine 5) (Cy5‐DOPE) were purchased from Avanti Polar Lipids (Alabaster, AL, USA).

### Construction of recombinant baculoviruses

Recombinant baculoviruses encoding PD‐1 mutant or Connexin 43 (Cx43)‐enhanced green fluorescence protein (EGFP) were constructed using the Bac‐to‐Bac Baculovirus Expression System, as described previously [22]. Briefly, pFastBac1 plasmid carrying the membrane protein sequence of interest was transformed into DH10Bac *Escherichia coli* containing Bacmid and a helper plasmid encoding a transposase gene. The target gene located between Tn7 transposon sequences was transposed into the Bacmid. Colonies containing the recombinant bacmid were identified by blue/white selection and the Bacmid was isolated using a PureLink HiPure Plasmid Miniprep Kit (Invitrogen). Sf9 cells were transfected with the recombinant Bacmid using Cellfectin II Reagent (Invitrogen) and incubated at 27 °C for 5 days. The supernatant containing P1 viruses was collected and the viral concentration was amplified three times. The viral titers were then determined using a BacPAK Baculovirus Rapid Titer Kit (Takara Bio USA, Inc., Palo Alto, CA, USA).

### Isolation of EVs

Sf9‐derived EVs were isolated using a previously reported method [22]. Briefly, 4.0 × 10^5^ Sf9 cells·mL^−1^ were maintained in Sf‐900 III SFM medium (Invitrogen) overnight at 27 °C. The budded virus (BV) suspension was added at multiplicity of infection of 0.5 and incubated at 27 °C for 96 h. The culture medium was centrifuged at 500 ***g*** for 5 min and 2000 ***g*** for 10 min at 4 °C, followed by 0.22‐µm filtration. The supernatant was ultra‐centrifuged at 100 000 ***g*** for 70 min at 4 °C and the resultant pellet was resuspended in phosphate‐buffered saline. The suspension was ultra‐centrifuged at 40 000 ***g*** for 30 min at 4 °C in a stepwise sucrose density gradient [10%, 15%, 20%, 25%, and 30% sucrose (w/v) in phosphate‐buffered saline buffer]. The upper fraction (containing EVs) and lower fraction (containing BVs) were collected separately. The EV protein concentration was estimated using a Pierce BCA Protein Assay Kit (Thermo Scientific, Waltham, MA, USA).

### Liposome preparation

DOPC, DOPS, NBD‐DOPE, Rho‐DOPE, and Cy5‐DOPE were mixed in chloroform into glass microtubes at various molar ratios. The solutions were evaporated under flowing argon gas, resulting in the formation of lipid film. The films were placed in a desiccator in vacuo overnight to completely remove chloroform. The film was hydrated by adding 250 µL of buffer [20 mm CH_3_COOH/CH_3_COONa (pH 4.5) or 10 mm Tris‐HCl (pH 7.5)] and incubated overnight at 27 °C. The suspension was extruded through a 100‐nm pore polycarbonate membrane using a mini‐extruder (Avanti Polar Lipids). The lipid concentration was measured using the Phospholipid C‐Test (Wako, Osaka, Japan), which quantifies choline produced by phospholipase D activity.

### Transmission electron microscopy (TEM)

Suspensions of EVs, BVs or hybrid EVs were placed on a copper grid coated with a formvar membrane for 5 min. After suspension removal, 1% phosphotungstic acid solution was placed on the grid for 5 min and then removed. Samples were observed using an HT7700‐TEM (Hitachi, Tokyo, Japan) at an accelerating voltage of 100 kV.

### Nanoparticle tracking analysis

The size distributions of EVs, liposomes, or hybrid EVs were measured using a NanoSight LM10 (NanoSight, Amesbury, UK). Samples were measured with a 532 nm wavelength laser at 25 °C and analyzed using nanosight nta, version 2.3 (NanoSight).

### Western blotting

EV samples solubilized with sodium dodecyl sulfate buffer were separated by 12.5% polyacrylamide gel and transferred to poly(vinylidene difluoride) membranes. The membranes were blocked with PVDF Blocking Reagent for Can Get Signal (Toyobo Co., Ltd, Osaka, Japan), then probed with primary antibody to gp64 (Santa Cruz Biotechnology), PD‐1 (Abcam, Cambridge, UK) or Cx43 (BD Transduction Laboratories, KY, USA) diluted at 1 : 1000 in Can Get Signal Solution 1 (Toyobo Co., Ltd) at 4 °C overnight. After they had been washed in Tris‐buffered saline with Tween, the bands were identified by reaction with horseradish peroxidase‐conjugated goat anti‐mouse IgG (Santa Cruz Biotechnology) diluted at 1 : 2000 in Can Get Signal Solution 2 (Toyobo Co., Ltd) at room temperature for 1 h. After a second round of washing in Tris‐buffered saline with Tween, the membranes were reacted with ECL Western Blotting Detection Reagents (GE Healthcare, Milwaukee, WI, USA) and the band signals were visualized using LAS‐4000 (GE Healthcare).

### Lipid mixing assay

For fluorescence resonance energy transfer (FRET)‐based lipid mixing analysis, liposomes were prepared at a 100 : 100 : 4 : 1 molar ratio of DOPC, DOPS, NBD‐DOPE, and Rho‐DOPE. The FRET liposomes (100 µm lipid) and PD‐1 EVs (60 µg·mL^−1^ protein) were mixed and incubated for 30 min at 27 °C under acidic conditions (pH 4.5) or neutral conditions (pH 7.5). In the antibody fusion inhibition test, PD‐1 EVs were pre‐incubated with anti‐gp64 antibody (sc‐65499; Santa Cruz Biotechnology) or mouse IgG2b isotype control antibody (401201; BioLegend, San Diego, CA, USA) diluted to a concentration of 0.2 or 2 µg·mL^−1^ in phosphate‐buffered saline at room temperature for 2 h. 7‐Nitro‐2‐1,3‐benzoxadiazol‐4‐yl (NBD) fluorescence intensity (excitation at 460 nm, emission at 535 nm) was measured using a FP8000 fluorescence spectrometer (Jasco, Tokyo, Japan). NBD fluorescence recovery was calculated using:

NBD fluorescence recovery (%) = (*F* – *F*_min_)/(*F*_max_ – *F*_min_)

where *F*_min_ represents the NBD fluorescence intensity of FRET liposomes before addition of PD‐1 EVs and *F*max represents the NBD fluorescence intensity of DOPC/DOPS/NBD‐DOPE (100 : 100 : 4 molar ratio) liposomes (100 µm lipid).

### Imaging flow cytometry (IFC)

For IFC analysis, PD‐1 EVs were stained with 5‐ or 6‐(*N*‐succinimidyloxycarbonyl)fluorescein 3′,6′‐diacetate (CFSE). CFSE was added to PD‐1 EV suspension to a final concentration of 62.8 µm, followed by incubation for 30 min at 37 °C. To remove free CFSE, the labeled PD‐1 EVs were washed using 100 000 NMWL Amicon Ultra Centrifugal Filters (Merck Millipore, Burlington, MA, USA). Liposomes were prepared at a 100 : 100 : 1 molar ratio of DOPC, DOPS, and Cy5‐DOPE. CFSE PD‐1 EVs and Cy5 liposomes, or Cx43‐EGFP EVs and Cy5 liposomes, were mixed at various ratios under acidic or neutral conditions and incubated for 30 min at 27 °C. Multispectral images of hybrid EVs were acquired using ImageStream^x^ MkII (Merck Millipore). Laser powers were set to maximum [488 nm : 200 mW; 642 nm : 150 mW; 785 nm (side scatter) : 70 mW]. Fluorescence signals were collected in channel 2 (CFSE or EGFP) or channel 5 (Cy5). Channels 1 and 6 were set to bright‐field and side scatter, respectively. The fluorescence intensities of 10 000 particles were acquired at 40× magnification and analyzed using ideas, version 6.2 (Merck Millipore).

### Statistical analysis

Experimental data were statistically evaluated using one‐way factorial analysis of variance followed by Tukey’s multiple comparisons test, or a two‐tailed Welch’s *t*‐test. An adjusted *P* < 0.05 was considered statistically significant. All statistical analyses were performed using prism, version 9 (GraphPad Software Inc., San Diego, CA, USA).

## Results and Discussion

### Lipid mixing assay of membrane fusion between liposomes and fusogenic EVs

**Fig. 1:**
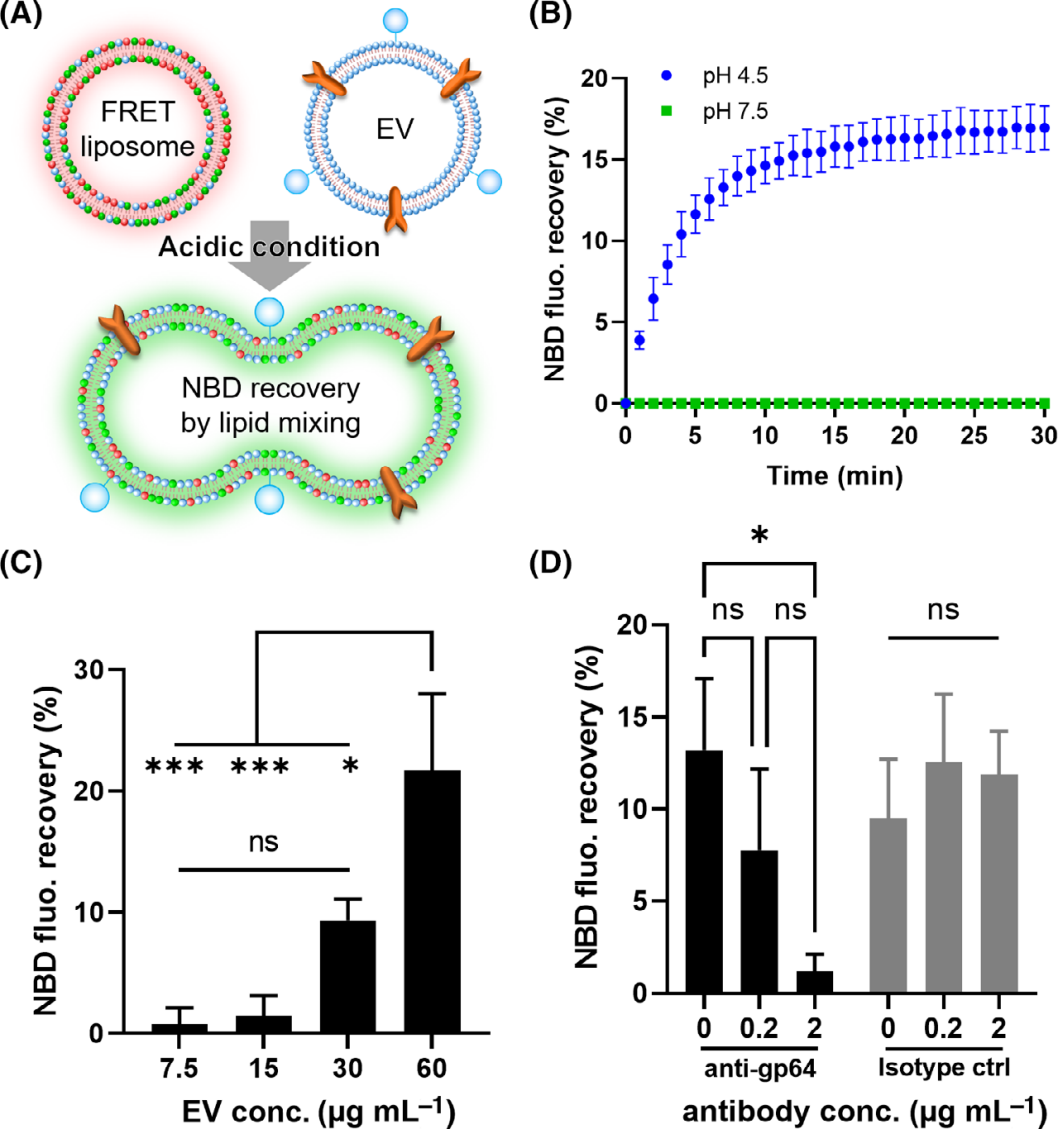
(A) Schematic illustration of FRET‐based lipid mixing assay using EVs and DOPC/DOPS/NBD‐DOPE/Rho‐DOPE liposomes. (B) Time course of NBD fluorescence recovery in a mixture of FRET liposomes (100 µm lipid) and PD‐1 EVs (60 µg·mL^−1^ protein) at pH 4.5 or 7.5. (C) Dependence of PD‐1 EV concentration on membrane fusion with FRET liposomes (100 µm lipid) at pH 4.5. (D) Inhibition analysis of membrane fusion of FRET liposomes (100 µm lipid) and PD‐1 EVs (60 µg·mL^−1^ protein) at pH 4.5 using an anti‐gp64 antibody or isotype control antibody. All results are expressed as the mean ± SD (*P < 0.05, ***P < 0.001, ns: nonsignificant, Tukey’s post‐hoc test, *n* = 3).

Ultra‐centrifugation was used to collect EVs from Sf9 insect cells that had been infected with recombinant baculoviruses. Basic characterization of Sf9‐derived EVs by TEM revealed successful isolation of vesicle‐like nanoparticles (Fig. S1A). Western blotting analysis confirmed the expression of the gp64 viral fusogenic protein on EVs (Fig. S1B). The EV fusion ability was evaluated by a lipid mixing assay based on FRET [27]. Membrane fusion of EVs and FRET liposomes containing 7‐nitro‐2‐1,3‐benzoxadiazol‐4‐yl (NBD) and rhodamine fluorescence was monitored on the basis of NBD fluorescence recovery by dilution of lipids (Fig. 1A). When FRET liposomes and EVs were mixed under acidic conditions (pH 4.5), NBD fluorescence recovered rapidly beginning 1 min after mixing; it eventually recovered to approximately 17% of the maximum fluorescence value (Fig. 1B, blue dots). By contrast, NBD fluorescence did not show any recovery under neutral conditions (Fig. 1B, green squares) or under acidic conditions in the absence of EVs (Fig. S2). Moreover, greater NBD fluorescence recovery efficiency was observed as the final concentration of EVs increased (Fig. 1C). To evaluate the specificity of gp64 fusion, an inhibition experiment was carried out using an anti‐gp64 antibody. As shown in Fig. 1D, the elimination of FRET under acidic conditions was suppressed depending on the concentration of anti‐gp64 antibody. By contrast, when an isotype control antibody was used, FRET elimination was not suppressed (Fig. 1D). These results indicated that the gp64 viral protein expressed on EVs could be activated under acidic conditions and induce membrane fusion with liposomes.

### Nanoparticle tracking analysis and TEM observation of hybrid EVs

**Fig. 2:**
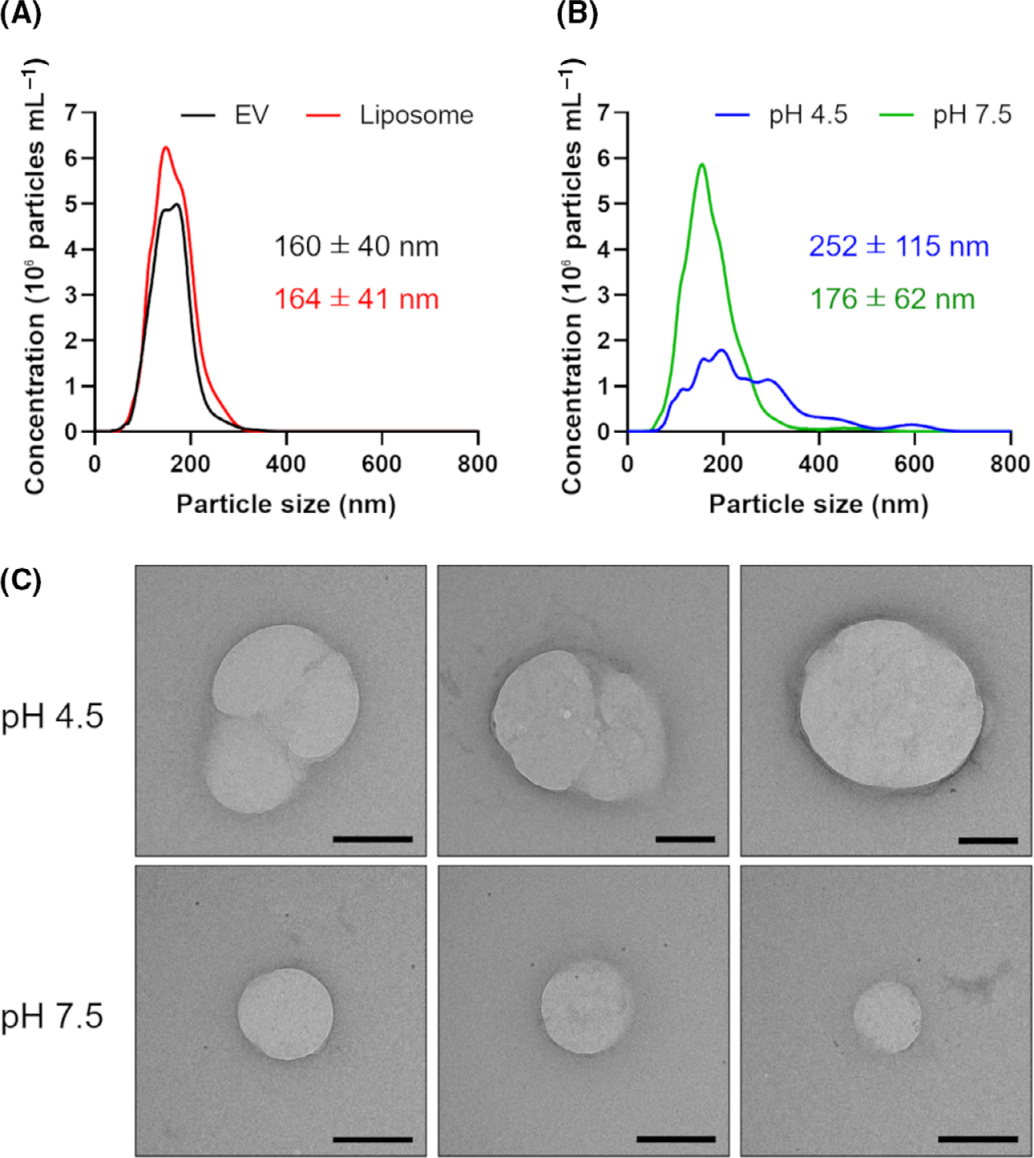
Size distributions of PD‐1 EVs and liposomes at pH 7.5 (A) and mixtures of PD‐1 EVs and liposomes at pH 4.5 or 7.5 (B) were determined by nanoparticle tracking analysis. All values are expressed as the mean ± SD. Shown are representative distributions from one of three independent experiments. (C) Representative TEM images of the fusion of PD‐1 EVs and liposomes at pH 4.5 or 7.5. Scale bars = 200 nm.

To investigate the fusion of EVs and liposomes, the particle size distribution of hybrid EVs was determined by nanoparticle tracking analysis. The respective mean particle sizes of EVs and liposomes before mixing were 160 nm and 164 nm (Fig. 2A). The particle sizes of EVs and liposomes mixed under acidic conditions increased and demonstrated a broad distribution (mean 252 nm) compared to EVs and liposomes mixed under neutral conditions (Fig. 2B). In addition, the number of detected particles decreased under acidic conditions. Because gp64 on EVs can mediate self‐fusion between EVs, the particle size distribution was measured after incubation of PD‐1 EVs under acidic conditions (Fig. S3). Compared with neutral conditions (Fig. 2A), the mean particle size increased slightly (184 nm). Although this may indicate self‐fusion between EVs, the change in the particle size distribution was not as drastic as that of the mixture of EVs and liposomes (Fig. 2B). The fusion between nanoparticles was directly observed by TEM (Fig. 2C). Under acidic conditions, hemifusion intermediates and fully fused large vesicles were observed. By contrast, under neutral conditions, no interparticle fusion was observed. There are several methods for directly visualizing the fusion of nanoparticles, such as electron microscopic observation of EVs and liposomes labeled with gold nanoparticles [18], or real‐time observation using high‐speed atomic force microscopy [28, 29]. Therefore, to confirm the formation of hybrid EVs, it is necessary to show the complexation of components of EVs and liposomes at the single‐particle level.

### Single‐particle analysis of membrane fusion by IFC

**Fig. 3:**
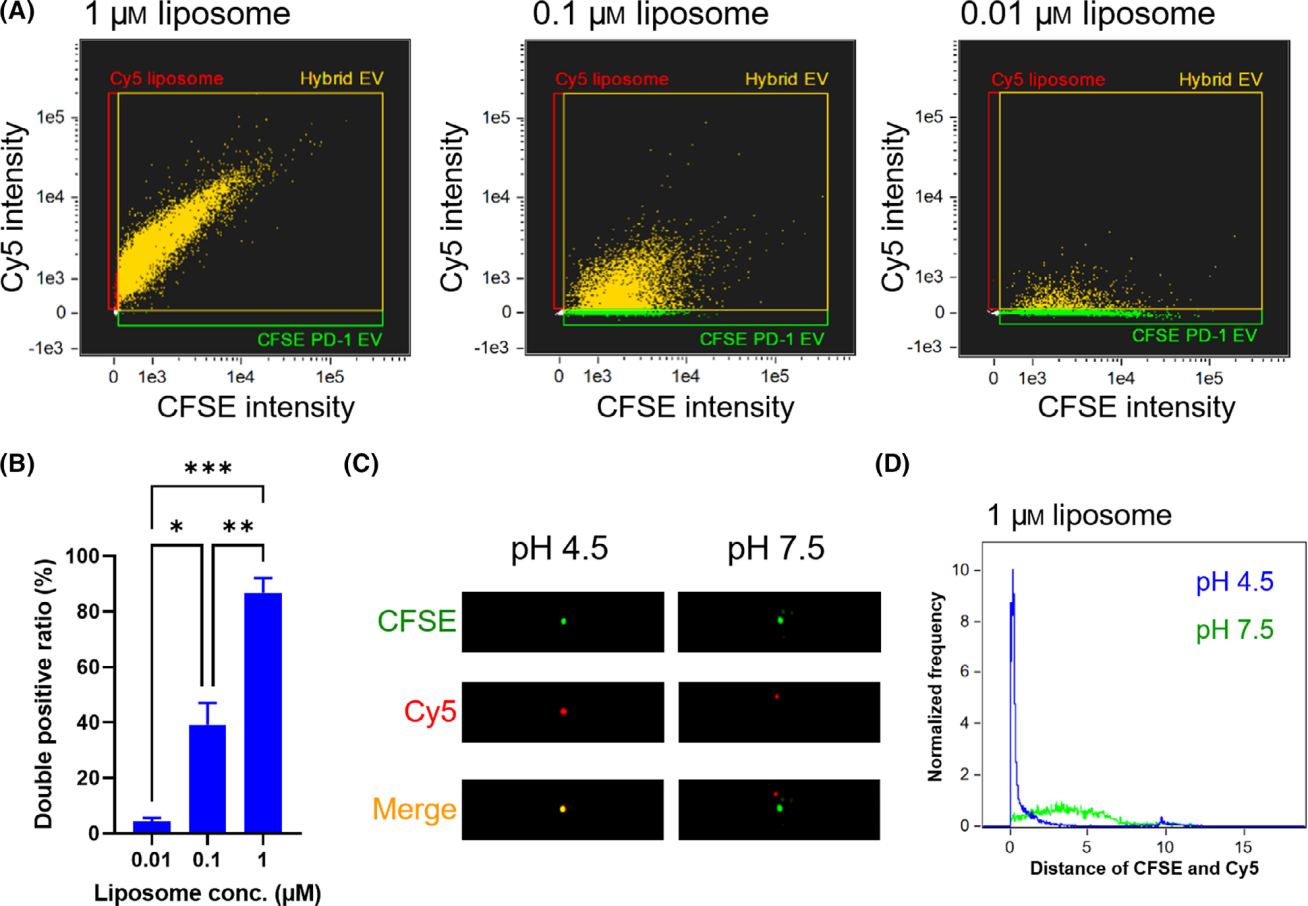
(A) Dot plots determined by IFC of mixtures of CFSE‐labeled PD‐1 EVs and Cy5 liposomes (1, 0.1, and 0.01 µm) under acidic conditions (green, CFSE‐single positive; red, Cy5‐single positive; yellow, CFSE and Cy5 double‐positive). (B) Proportions of CFSE and Cy5 double‐positive particles under acidic conditions. The result is expressed as the mean ± SE (*P < 0.05, **P < 0.01, ***P < 0.001, Tukey’s post‐hoc test, *n* = 3). (C) Representative fluorescence images determined by IFC of double‐positive particles prepared by fusion of PD‐1 EVs and 1 µm liposomes under acidic conditions. (D) The distance of CFSE and Cy5 fluorescence spots was represented as the number of pixels between the centroids of both fluorescence regions.

Next, the complexation of EVs and liposomes at the single‐particle level was evaluated using IFC, a high‐throughput analysis method that combines the functions of flow cytometry and fluorescence microscopy. Because of its high sensitivity, IFC enables fluorescence analysis of EVs at the single‐particle level, which has been difficult thus far [30, 31]. Sf9‐derived EVs were labeled with CFSE to evaluate their complexation with Cy5‐labeled liposomes. CFSE serves to label proteins inside of EVs; it fluoresces following hydrolysis by esterase in EVs. CFSE‐labeled EVs were successfully detected by IFC, whereas they were absent in control groups (Fig. S4). CFSE‐labeled EVs and Cy5 liposomes were mixed under various conditions and particle populations were analyzed by IFC using gating strategy shown in Fig. S5. Under acidic conditions, there was a greater proportion of particles in the CFSE and Cy5 double‐positive region, following changes in the EV‐liposome ratio (Fig. 3A,B). By contrast, under neutral conditions, the proportion of particles present in the double‐positive region was low (Fig. S6, S7). The proportion of double‐positive particles is considered the fusion efficiency at the single‐particle level. Fig. 3C,D shows co‐localization of CFSE and Cy5 in hybrid EVs prepared from 1 µm liposomes under acidic conditions. Under acidic conditions, most of the CFSE and Cy5 fluorescence spots detected in hybrid EV region overlapped, whereas under neutral conditions, there was little overlap (Fig. 3D). Furthermore, Cy5 fluorescence intensity in the double‐positive region increased under acidic conditions, depending on liposome concentration (Fig. S8). This result indicates that the surface modification of hybrid EVs at single‐particle level can be controlled by changing the EV‐liposome ratio.

**Fig. 4:**
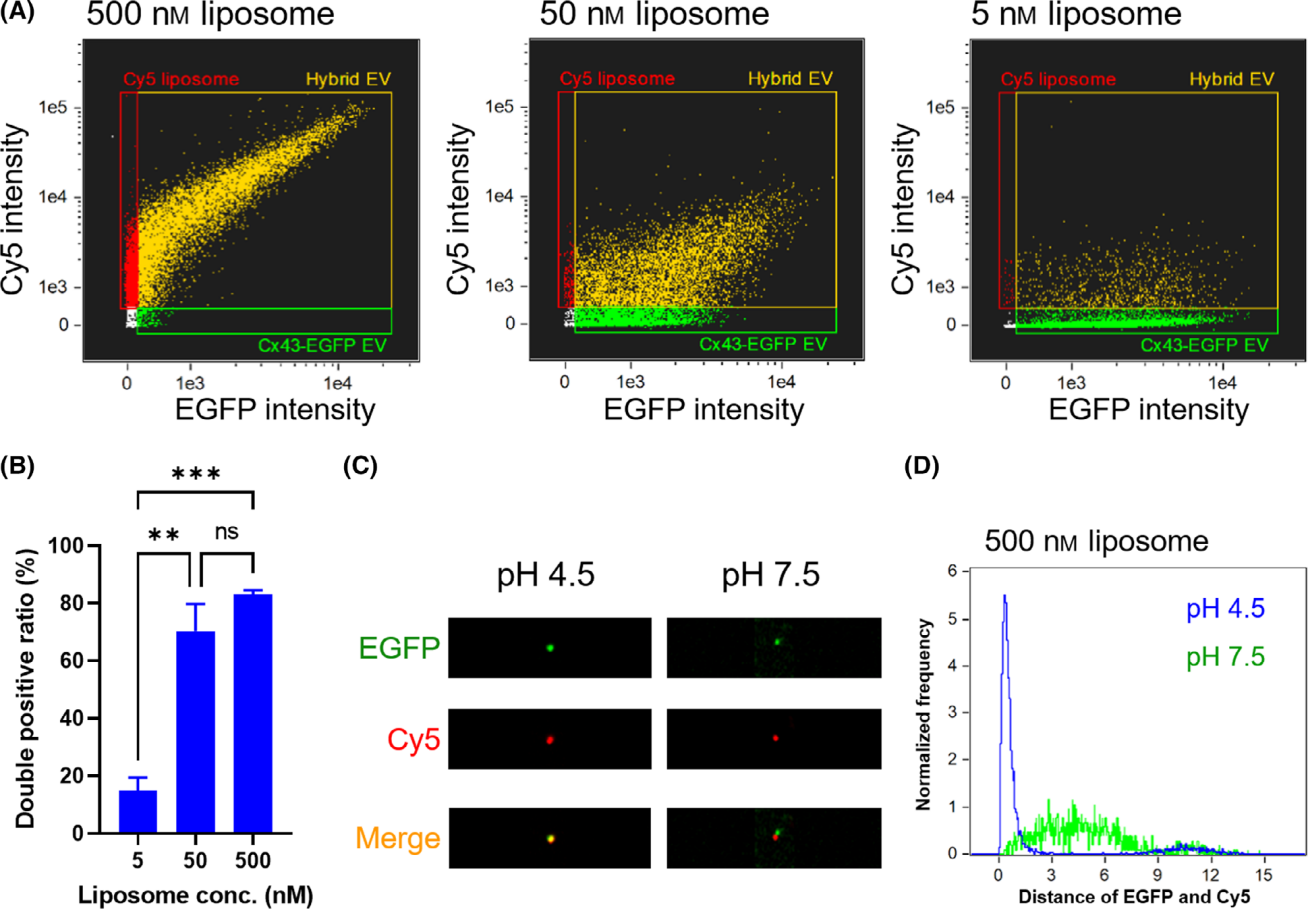
(A) Dot plots determined by IFC of mixtures of Cx43‐EGFP EVs and Cy5 liposomes (500, 50, and 5 nm) under acidic conditions (green, EGFP‐single positive; red, Cy5‐single positive; yellow, EGFP and Cy5 double‐positive). (B) Proportions of EGFP and Cy5 double‐positive particles under acidic conditions. The result is expressed as the mean ± SE (**P < 0.01, ***P < 0.001, ns: nonsignificant, Tukey’s post‐hoc test, *n* = 3). (C) Representative fluorescence images determined by IFC of double‐positive particles prepared by fusion of Cx43‐EGFP EVs and 500 nm liposomes under acidic conditions. (D) The distance of EGFP and Cy5 fluorescence spots was represented as the number of pixels between the centroids of both fluorescence regions.

Finally, Sf9‐derived EVs expressing enhanced green fluorescence protein‐conjugated Cx43 (i.e. Cx43‐EGFP) were used to evaluate the co‐localization of EV and liposome membranes. Cx43 is a four‐pass transmembrane protein and a component of gap junctions [25]. Cx43‐EGFP EVs were isolated from Sf9 cells infected with recombinant baculoviruses; the expression of gp64 and Cx43 on Cx43‐EGFP EVs was analyzed by western blotting (Fig. S1B). Membrane fusion of Cx43‐EGFP EVs and Cy5 liposomes was visualized at the single‐particle level by IFC. The particle populations shifted toward the EGFP and Cy5 double‐positive region depending on changes in the EV‐liposome ratio under acidic conditions (Fig. 4A,B; Fig. S9, S10). EGFP and Cy5 fluorescence spots were co‐localized under acidic conditions, but not under neutral conditions (Fig. 4C,D). Moreover, liposome concentration‐dependent Cy5 fluorescence transfer to Cx43‐EGFP EVs suggested the potential to freely perform surface engineering of hybrid EVs (Fig. S11). These results suggest that EVs and small liposomes fused under acidic conditions to form hybrid EV particles in which membrane protein components were transferred.

Though the method of fusion between EVs and liposomes has been verified by various approaches, some problems such as contamination of exogenous materials (e.g. polyethylene glycol) and a long fusion reaction time (e.g. 2–12 h) remain [16, 18]. By contrast, the fusion method in the present study is a very simple and rapid process of incubation for 30 min under acidic conditions. Furthermore, single‐particle analysis of membrane fusion by IFC enabled the determination of detailed EV‐liposome fusion efficiency and surface composition of hybrid EVs, which have not been clarified so far.

## Conclusions

In conclusion, we have developed hybrid EVs by membrane fusion of small liposomes and EVs equipped with baculoviral fusogenic proteins. The gp64‐mediated fusion of EVs and liposomes was analyzed at the single‐particle level, which revealed controlled introduction of liposome components into EVs. The achievement of highly efficient EV‐liposome membrane fusion and the evaluation methods used in the present study will support further applications of EV engineering in biomedicine. We expect that the hybrid EVs will serve as new drug delivery carriers that achieve cell membrane fusion by means of fusogenic gp64 and active targeting by means of functional membrane proteins such as PD‐1 and Cx43.

## Conflicts of interest

The authors declare that they have no conflicts of interest.

## Author contributions

RI, SY, and KA designed the study. RI performed experiments, analyzed and interpreted the data, and wrote the manuscript. SY, SS, YS, and KA interpreted the data and supervised the study. KA and RI acquired the research funding. All authors discussed the results and approved the final version of the manuscript submitted for publication.

## Supporting information


**Fig. S1**. Basic characterization of Sf9‐derived samples. (A) Morphologies of PD‐1 EVs (upper image) and PD‐1 BVs (budded viruses) (lower image) were observed by TEM. Scale bars = 200 nm. (B) Western blotting analysis of PD‐1 EVs and Cx43‐EGFP EVs using anti‐gp64, anti‐PD‐1 or anti‐Cx43 antibodies.
**Fig. S2**. Time course of NBD fluorescence recovery of FRET liposomes (100 µm lipid) diluted with pH 4.5 buffer. All values are expressed as the mean ± SD (*n* = 3).
**Fig. S3**. Size distributions of PD‐1 EVs and liposomes at pH 4.5 were determined by nanoparticle tracking analysis. Shown are representative distributions from one of three independent experiments. All values are expressed as the mean ± SD.
**Fig. S4**. Dot plots and representative fluorescence images determined by IFC of CFSE‐labeled EVs (A) and control samples (B–D). Addition of CFSE to bovine serum albumin (BSA) protein with esterase activity showed very high background fluorescence, although ultra‐filtration purification removed most background fluorescence.
**Fig. S5**. Gating strategy for detection of fluorescence nanoparticles by IFC. Plots were obtained in the 1 µm liposomes (pH 4.5) condition; the gating process was similar for other conditions. (A) Removal of speed beads using channels 1 (bright‐field) and 6 (side scatter). (B) Removal of fluorescent noise for channels 2 and 5. Finally, 10 000 particles were acquired and analyzed in the R2 region.
**Fig. S6**. Dot plots determined by IFC of mixtures of CFSE‐labeled PD‐1 EVs and Cy5 liposomes (1, 0.1, and 0.01 µm) under neutral conditions (green, CFSE‐single positive; red, Cy5‐single positive; yellow, CFSE and Cy5 double‐positive).
**Fig. S7**. (A) Proportions of CFSE and Cy5 double‐positive particles prepared by fusion of PD‐1 EVs and 1 µm Cy5 liposomes at pH 4.5 or 7.5 (**P < 0.01, two‐tailed Welch’s *t*‐test, *n* = 3). (B) Proportions of CFSE and Cy5 double‐positive particles at pH 7.5 (*P < 0.05, **P < 0.01, ns: nonsignificant, Tukey’s post‐hoc test, *n* = 3). All results are expressed as the mean ± SE.
**Fig. S8**. (A) Median Cy5 intensity of hybrid EVs (CFSE and Cy5 double‐positive) prepared under acidic conditions. The result is expressed as the mean ± SE (*P < 0.05, **P < 0.01, ns: nonsignificant, Tukey’s post‐hoc test, *n* = 3). (B) Cy5 fluorescence histogram of the mixture of CFSE PD‐1 EVs and Cy5 liposomes (1 µm) in the double‐positive region.
**Fig. S9**. Dot plots determined by IFC of mixtures of Cx43‐EGFP EVs and Cy5 liposomes (500, 50, and 5 nm) under neutral conditions (green, EGFP‐single positive; red, Cy5‐single positive; yellow, EGFP and Cy5 double‐positive).
**Fig. S10**. (A) Proportions of EGFP and Cy5 double‐positive particles prepared by fusion of Cx43‐EGFP EVs and 500 nm Cy5 liposomes at pH 4.5 or 7.5 (****P < 0.0001, two‐tailed Welch’s *t*‐test, *n* = 3). (B) Proportions of EGFP and Cy5 double‐positive particles at pH 7.5 (ns: nonsignificant, Tukey’s post‐hoc test, *n* = 3). All results are expressed as the mean ± SE.
**Fig. S11**. (A) Median Cy5 intensity of hybrid EVs (EGFP and Cy5 double‐positive) prepared under acidic conditions. The result is expressed as the mean ± SE (**P < 0.01, ns: nonsignificant, Tukey’s as mean test, *n* = 3). (B) Cy5 fluorescence histogram of the mixture of Cx43‐EGFP EVs and Cy5 liposomes (500 nm) in the double‐positive region.

## Data Availability

The data that support the findings of the present study are provided within the figures.
