# Supplementary material for: Development and single‐particle analysis of hybrid extracellular vesicles fused with liposomes using viral fusogenic proteins
Source: FEBS Open Bio. 2022 Apr 30;12(6):1178–87. doi: 10.1002/2211-5463.13406 (PMC9157406; doi:10.1002/2211-5463.13406)
Supplement: Supplementary file 1 — Fig. S1. Basic characterization of Sf9‐derived samples. (A) Morphologies of PD‐1 EVs (upper image) and PD‐1 BVs (budded viruses) (lower image) were observed by TEM. Scale bars = 200 nm. (B) Western blotting analysis of PD‐1 EVs and Cx43‐EGFP EVs using anti‐gp64, anti‐PD‐1 or anti‐Cx43 antibodies. Fig. S2. Time course of NBD fluorescence recovery of FRET liposomes (100 µm lipid) diluted with pH 4.5 buffer. All values are expressed as the mean ± SD (n = 3). Fig. S3. Size distributions of PD‐1 EVs and liposomes at pH 4.5 were determined by nanoparticle tracking analysis. Shown are representative distributions from one of three independent experiments. All values are expressed as the mean ± SD. Fig. S4. Dot plots and representative fluorescence images determined by IFC of CFSE‐labeled EVs (A) and control samples (B–D). Addition of CFSE to bovine serum albumin (BSA) protein with esterase activity showed very high background fluorescence, although ultra‐filtration purification removed most background fluorescence. Fig. S5. Gating strategy for detection of fluorescence nanoparticles by IFC. Plots were obtained in the 1 µm liposomes (pH 4.5) condition; the gating process was similar for other conditions. (A) Removal of speed beads using channels 1 (bright‐field) and 6 (side scatter). (B) Removal of fluorescent noise for channels 2 and 5. Finally, 10 000 particles were acquired and analyzed in the R2 region. Fig. S6. Dot plots determined by IFC of mixtures of CFSE‐labeled PD‐1 EVs and Cy5 liposomes (1, 0.1, and 0.01 µm) under neutral conditions (green, CFSE‐single positive; red, Cy5‐single positive; yellow, CFSE and Cy5 double‐positive). Fig. S7. (A) Proportions of CFSE and Cy5 double‐positive particles prepared by fusion of PD‐1 EVs and 1 µm Cy5 liposomes at pH 4.5 or 7.5 (**P < 0.01, two‐tailed Welch’s t‐test, n = 3). (B) Proportions of CFSE and Cy5 double‐positive particles at pH 7.5 (*P < 0.05, **P < 0.01, ns: nonsignificant, Tukey’s post‐hoc test, n = 3). All results ar [file FEB4-12-1178-s001.docx]

**Supporting Information**

Development and single-particle analysis of hybrid extracellular vesicles fused with liposomes using viral fusogenic proteins

Raga Ishikawa^†^, Shosuke Yoshida^††^, Shin-ichi Sawada, Yoshihiro Sasaki, Kazunari Akiyoshi*

Department of Polymer Chemistry, Graduate School of Engineering, Kyoto University, Katsura, Nishikyo-ku, Kyoto 615-8510, Japan

**Present Addresses**

^†^ Department of Environmental Engineering, Graduate School of Engineering, Kyoto University, Katsura, Nishikyo-ku, Kyoto 615-8540, Japan

^††^ Division of Biological Science, Nara Institute of Science and Technology, 8916-5 Takayama-cho, Ikoma, Nara 630-0192, Japan

*Corresponding Author

Prof. Dr. Kazunari Akiyoshi

E-mail address: akiyoshi@bio.polym.kyoto-u.ac.jp

**
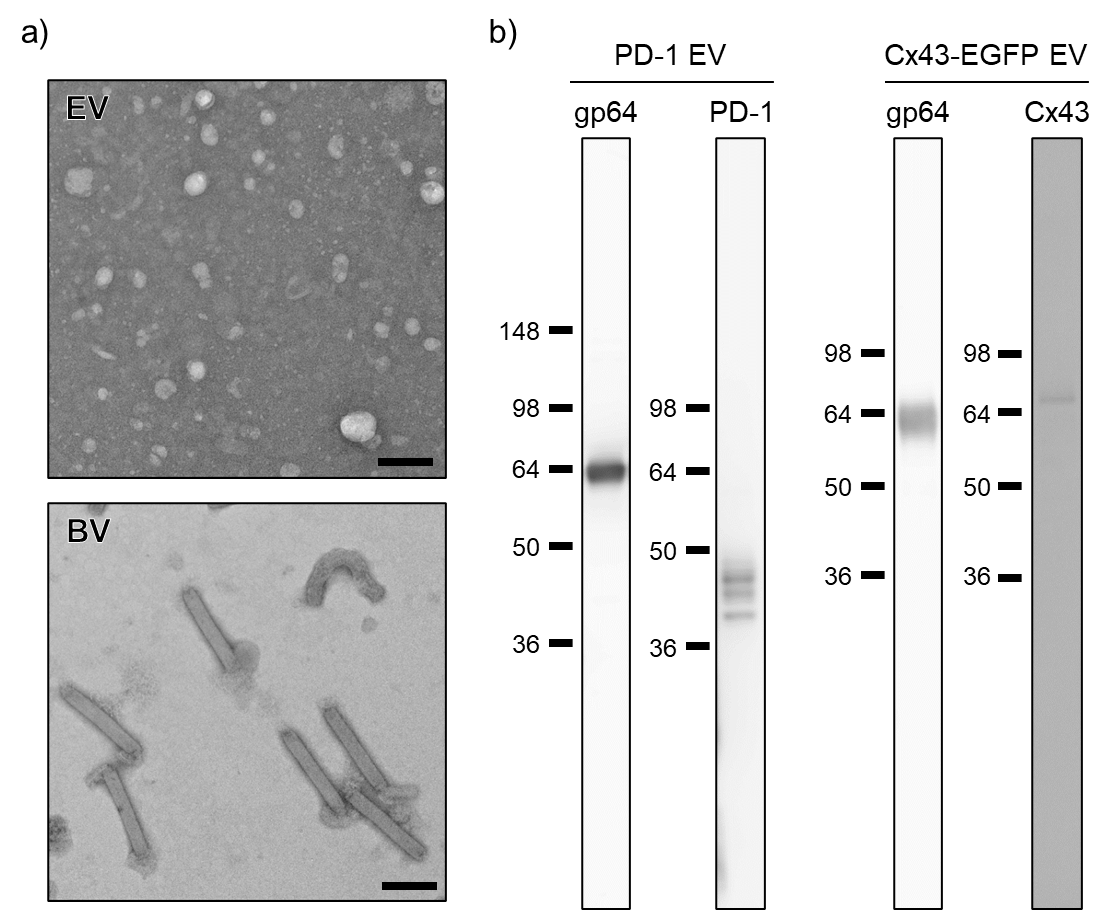
**

**Figure S1.** Basic characterization of Sf9-derived samples. (a) Morphologies of PD-1 EVs (upper image) and PD-1 BVs (budded viruses) (lower image) were observed by TEM. Scale bars, 200 nm. (b) Western blotting analysis of PD-1 EVs and Cx43-EGFP EVs using anti-gp64, anti-PD-1, or anti-Cx43 antibodies.

**Figure S2.** Time course of NBD fluorescence recovery of FRET liposomes (100 µM lipid) diluted with pH 4.5 buffer. All values are expressed as mean ± SD (n = 3).


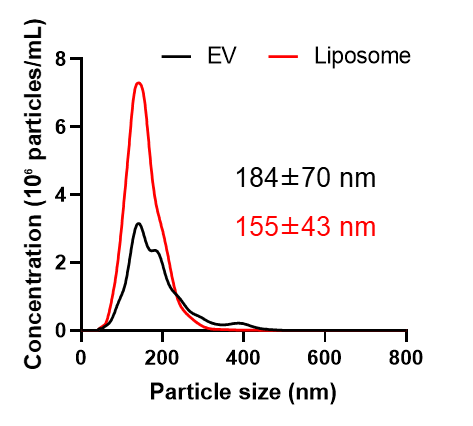


**Figure S3.** Size distributions of PD-1 EVs and liposomes at pH 4.5 were determined by nanoparticle tracking analysis. Shown are representative distributions from one of three independent experiments. All values are expressed as mean ± SD.


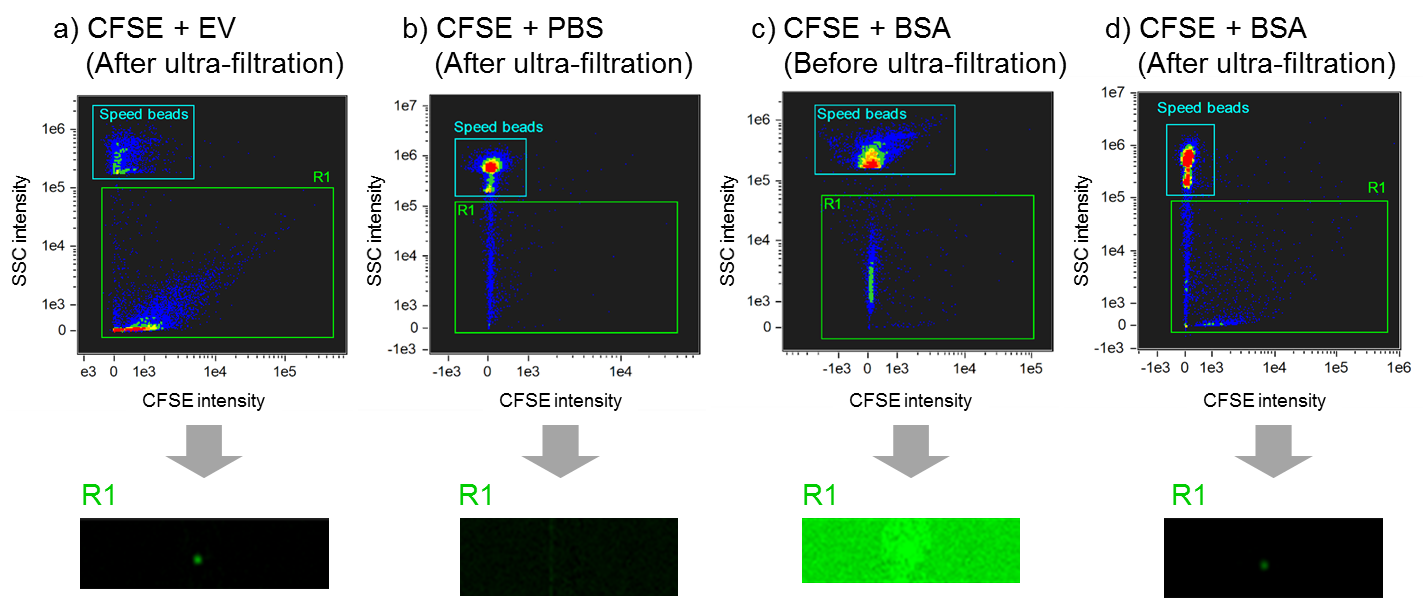


**Figure S4.** Dot plots and representative fluorescence images determined by IFC of CFSE-labeled EVs (a) and control samples (b–d). Addition of CFSE to bovine serum albumin (BSA) protein with esterase activity showed very high background fluorescence, although ultra-filtration purification removed most background fluorescence.


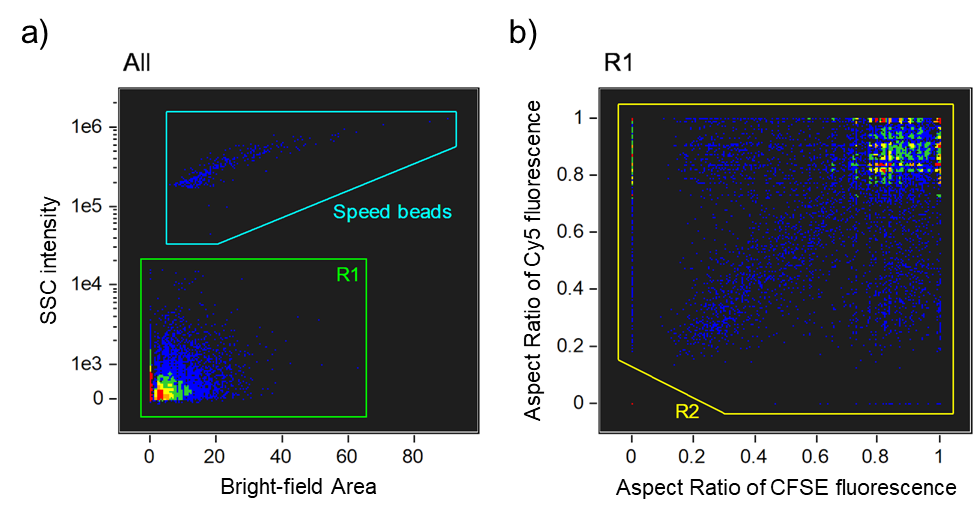


**Figure S5.** Gating strategy for detection of fluorescence nanoparticles by IFC. Plots were obtained in the 1 µM liposomes (pH 4.5) condition; the gating process was similar for other conditions. (a) Removal of speed beads using channels 1 (bright-field) and 6 (side scatter). (b) Removal of fluorescent noise for channels 2 and 5. Finally, 10,000 particles were acquired and analyzed in the R2 region.


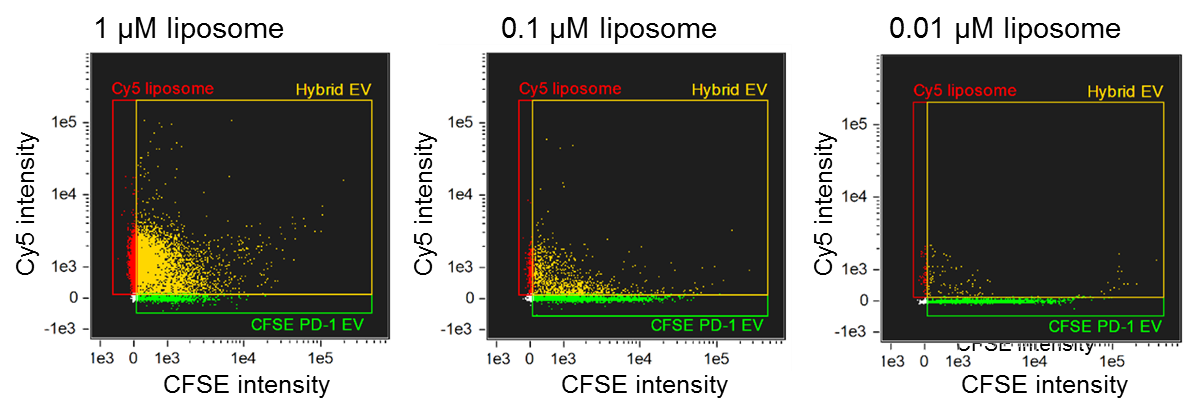


**Figure S6.** Dot plots determined by IFC of mixtures of CFSE-labeled PD-1 EVs and Cy5 liposomes (1, 0.1, and 0.01 µM) under neutral conditions (green, CFSE-single positive; red, Cy5-single positive; yellow, CFSE and Cy5 double-positive).


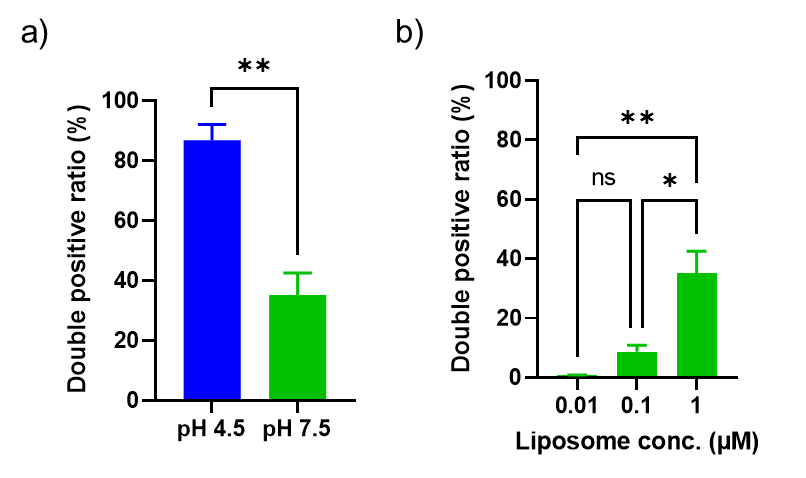


**Figure S7.** (a) Proportions of CFSE and Cy5 double-positive particles prepared by fusion of PD-1 EVs and 1 µM Cy5 liposomes at pH 4.5 or 7.5 (**p < 0.01, two-tailed Welch’s *t*-test, n= 3). (b) Proportions of CFSE and Cy5 double-positive particles at pH 7.5 (*p < 0.05, **p < 0.01, ns: nonsignificant, Tukey’s post hoc test, n = 3). All results are expressed as mean ± SE.


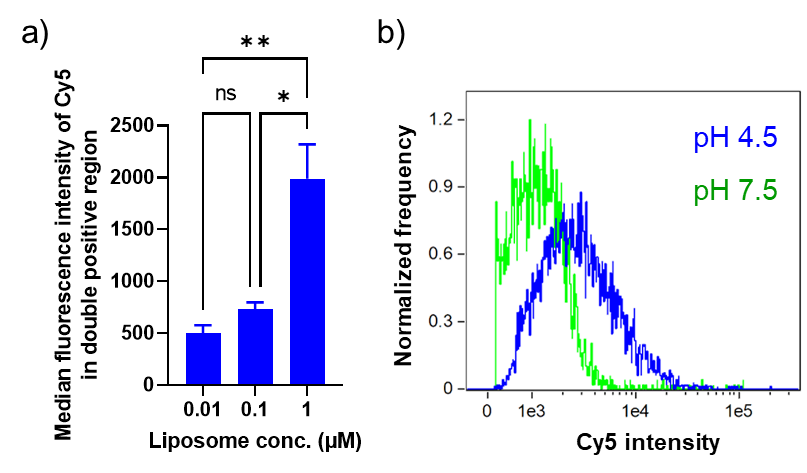


**Figure S8.** (a) Median Cy5 intensity of hybrid EVs (CFSE and Cy5 double-positive) prepared under acidic conditions. The result is expressed as mean ± SE (*p < 0.05, **p < 0.01, ns: nonsignificant, Tukey’s post hoc test, n = 3). (b) Cy5 fluorescence histogram of the mixture of CFSE PD-1 EVs and Cy5 liposomes (1 µM) in the double-positive region.


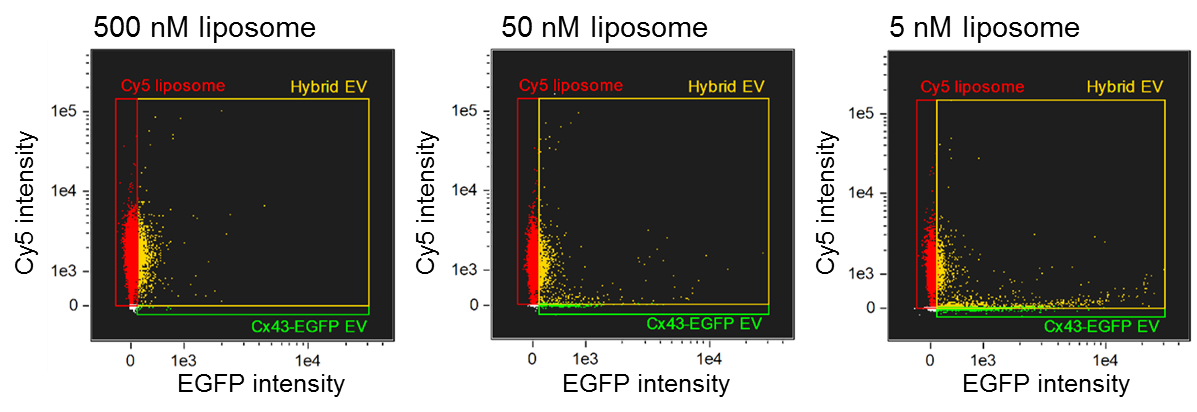


**Figure S9.** Dot plots determined by IFC of mixtures of Cx43-EGFP EVs and Cy5 liposomes (500, 50, and 5 nM) under neutral conditions (green, EGFP-single positive; red, Cy5-single positive; yellow, EGFP and Cy5 double-positive).

**
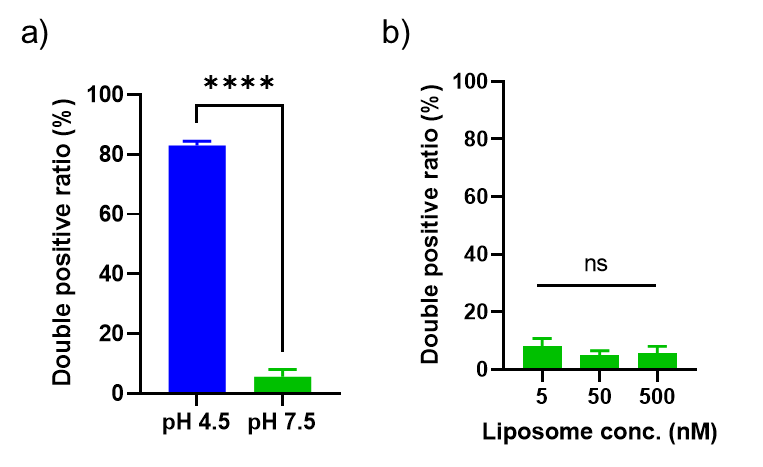
**

**Figure S10.** (a) Proportions of EGFP and Cy5 double-positive particles prepared by fusion of Cx43-EGFP EVs and 500 nM Cy5 liposomes at pH 4.5 or 7.5 (****p < 0.0001, two-tailed Welch’s *t*-test, n= 3). (b) Proportions of EGFP and Cy5 double-positive particles at pH 7.5 (ns: nonsignificant, Tukey’s post hoc test, n = 3). All results are expressed as mean ± SE.


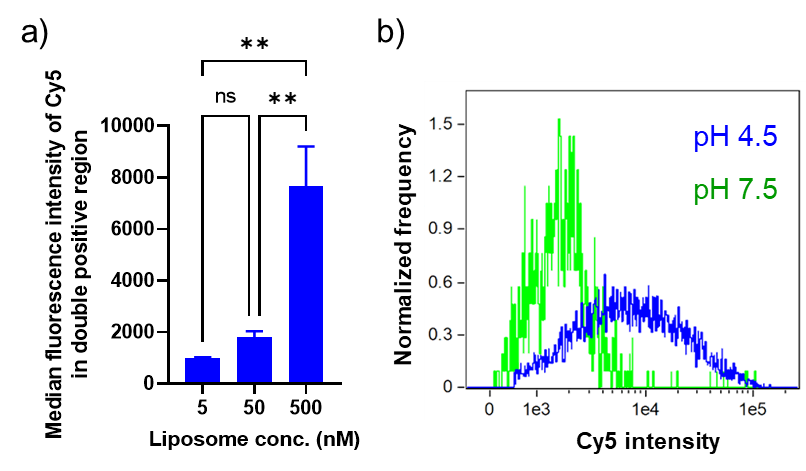


**Figure S11.** (a) Median Cy5 intensity of hybrid EVs (EGFP and Cy5 double-positive) prepared under acidic conditions. The result is expressed as mean ± SE (**p < 0.01, ns: nonsignificant, Tukey’s post hoc test, n = 3). (b) Cy5 fluorescence histogram of the mixture of Cx43-EGFP EVs and Cy5 liposomes (500 nM) in the double-positive region.
